# Multiple Sclerosis and SARS-CoV-2: Has the Interplay Started?

**DOI:** 10.3389/fimmu.2021.755333

**Published:** 2021-09-27

**Authors:** Gianmarco Bellucci, Virginia Rinaldi, Maria Chiara Buscarinu, Roberta Reniè, Rachele Bigi, Giulia Pellicciari, Emanuele Morena, Carmela Romano, Antonio Marrone, Rosella Mechelli, Marco Salvetti, Giovanni Ristori

**Affiliations:** ^1^ Centre for Experimental Neurological Therapies (CENTERS), Department of Neurosciences, Mental Health and Sensory Organs, Sapienza University of Rome, Rome, Italy; ^2^ Neuroimmunology Unit, Istituto di Ricovero e Cura a Carattere Scientifico (IRCCS) Fondazione Santa Lucia, Rome, Italy; ^3^ Istituto di Ricovero e Cura a Carattere Scientifico (IRCCS) San Raffaele Pisana, Rome, Italy; ^4^ San Raffaele Roma Open University, Rome, Italy; ^5^ Istituto di Ricovero e Cura a Carattere Scientifico (IRCCS) Istituto Neurologico Mediterraneo Neuromed, Pozzilli, Italy

**Keywords:** multiple sclerosis, SARS-CoV-2, COVID-19, virus-host interactions, neuroinflammation, vaccine, virus, EBV—Epstein-Barr Virus

## Abstract

Current knowledge on Multiple Sclerosis (MS) etiopathogenesis encompasses complex interactions between the host’s genetic background and several environmental factors that result in dysimmunity against the central nervous system. An old-aged association exists between MS and viral infections, capable of triggering and sustaining neuroinflammation through direct and indirect mechanisms. The novel Coronavirus, SARS-CoV-2, has a remarkable, and still not fully understood, impact on the immune system: the occurrence and severity of both acute COVID-19 and post-infectious chronic illness (long COVID-19) largely depends on the host’s response to the infection, that echoes several aspects of MS pathobiology. Furthermore, other MS-associated viruses, such as the Epstein-Barr Virus (EBV) and Human Endogenous Retroviruses (HERVs), may enhance a mechanistic interplay with the novel Coronavirus, with the potential to interfere in MS natural history. Studies on COVID-19 in people with MS have helped clinicians in adjusting therapeutic strategies during the pandemic; similar efforts are being made for SARS-CoV-2 vaccination campaigns. In this Review, we look over 18 months of SARS-CoV-2 pandemic from the perspective of MS: we dissect neuroinflammatory and demyelinating mechanisms associated with COVID-19, summarize pathophysiological crossroads between MS and SARS-CoV-2 infection, and discuss present evidence on COVID-19 and its vaccination in people with MS.

## Introduction

Multiple Sclerosis (MS) is a chronic, immune-mediated disease of the Central Nervous System (CNS) characterized by focal demyelination and neurodegeneration. It is the leading cause of non-traumatic disability in young people, as its onset typically occurs between 20 and 40 years of age. Although the cause(s) of MS remain unknown, a multifactorial model is widely accepted: diverse “environmental” factors may trigger the immune attack and disease progression in genetically susceptible individuals (1–3).

Solid preclinical and clinical data support the role of viral infections in MS etiology. It is plausible that MS neurodegenerative and inflammatory processes start years before the epiphany of radiological and clinical manifestations (4–7). In this context, viral agents are good candidates to uncover the disease, as they are capable to precipitate the dysimmune process *via* multiple mechanisms: molecular mimicry, bystander activation, epitope spreading, autoreactive immune cell survival and immortalization, regulome modifications (3, 8–10). Thus, the selection and global spread of a novel human-infecting virus represents an experimental challenge to study virus-associated diseases, including MS. The research rush enlightened by SARS-CoV-2 diffusion may help to elucidate the “known unknowns” of virus-host interactions in immune-mediated diseases and accelerate drug tailoring. Furthermore, the novel coronavirus itself—with its pronounced effect on the host’s immune system—may be directly involved in development, or divert the natural history, of dysimmune diseases, depicting a new epidemiological map in the forthcoming years. In this review we will synopsize present knowledge on SARS-COV-2 and neuroinflammation, focusing on the crossroads of SARS-CoV-2 infection and MS physiopathology; we will also reassess current evidence on COVID-19 and SARS-CoV-2 vaccines in people with MS and discuss a potential inference in MS disease course.

## SARS-CoV-2 and Neuroinflammation

Human-infecting Coronaviruses include four mild pathogenic strains (229E, NL63, OC43, and HKU1), which cause up to a third of common cold cases, and three highly virulent agents: SARS-CoV-1, MERS, and SARS-CoV-2. The latter pathogen has emerged in China in late 2019 and has quickly become pandemic, due to its extreme infectivity (11). In the acute phase, most of infected patients have no or mild symptoms, but up to 15% cases require hospitalization for a severe interstitial pneumonia, which has caused, to date, more than 4 million deaths.

COVID-19 is a multifaceted and unpredictable syndrome whose outcomes are primarily determined by the host’s immune response. Compared to other respiratory viruses, SARS-CoV-2 elicits a stronger, perduring, auto-aggressive inflammatory response (12), fueled by a massive cytokine release, causing coagulation dysfunction and multiorgan failure (13) in severe cases. The aberrant immune activation is clear-cut in children, who can suffer of a virus-induced multisystem inflammatory syndrome (MIS-C) with prominent autoimmune components (14, 15).

A SARS-CoV-2 neuroinvasive potential has been reported (16) as for other Coronaviruses (17), that have also been isolated in brains and cerebrospinal fluid (CSF) of MS patients with significant frequency (18, 19). Blood-brain barrier pericytes and astrocytes may represent SARS-CoV-2 entry points (20). Neurological symptoms are reported in more than 80% cases during disease history and, opposite to the respiratory distress syndrome, are more frequent in young people (21). Ischemic stroke may be a SARS-CoV-2 specific presentation, especially in people under 50 years of age (22, 23) as a consequence of the hypercoagulation state induced by systemic inflammation. Besides non-specific symptoms (headache, confusional states, dizziness, etc.) that are common complications of viral infections and critical illnesses (24), most of neuro-COVID manifestations share an immune-mediate substrate. In fact, brain lesions associated to COVID-19 reflect both vascular and demyelinating etiologies and are mainly imputable to the intense immune activation with massive neurotoxic cytokines production (in particular, IL1-beta and IL6) (25), rather than a direct infectious cytopathic effect. A prominent example comes from a neuropathological description of vascular and acute disseminated encephalomyelitis (ADEM)-like demyelinating pathology in a COVID-19 patient (26).

Omics studies on brain samples from COVID-19 patients are pinpointing the neuroinflammatory mechanisms underlying neurological involvement. Yang et al. (27) performed single-nucleus transcriptomics and immunohistochemistry of cortex and choroid plexus of eight COVID-19 brains. First, they highlighted that, albeit all major brain cell types resulted affected, glial cells displayed a pro-inflammatory, disease-specific signature. Second, they underscored significant brain barrier inflammation in the choroid plexus, a strategic interface between peripheral blood and cerebrospinal fluid. In COVID-19 brains, choroid barrier cells and glia limitans released chemokines toward brain parenchyma and promoted complement activation, fueling neuroinflammation and neural damages. A similar mechanism was described in MS, where an activated choroid epithelium in response to peripheral inflammation acts as a gateway for brain-homing, pathogenic B and T lymphocytes (28–30). Likely, infiltrating T cells were found in COVID-19 brain parenchyma, in the absence of SARS-CoV-2 RNA transcripts, suggesting an aberrant CNS attack by peripheral immune effectors favored by BBB disruption.

Comparison of transcriptome signatures between COVID-19 brains and chronic CNS diseases (including MS) revealed a significant intersection of dysregulated genes, particularly in glial cells. A notable example comes from *RIPK1*, whose overactivation in microglia and astrocytes contributes to MS pathology, particularly in the progressive disease (31). Moreover, COVID-19 differentially expressed genes are enriched with GWAS-associated variants of complex neurological traits, suggesting a possible interaction of the novel Coronavirus to the initiation or perpetuation of CNS disorders in individuals who are at risk (27).

Schwabenland et al. (32) found extensive T CD8 cell infiltration, microgliosis, and increased axonal damage in COVID-19 comparable to that seen in long-term MS patients. A disease-specific cluster of CD8+ lymphocytes was present in the perivascular space, causing vascular immunopathology and BBB disruption. Indeed, endothelial cells with viral proteins in the cytoplasm colocalize with innate and adaptive immune effectors, vascular pathology, and axonal damage. The crosstalk between cytotoxic lymphocytes and microglial cells peaks in the formation of microanatomical niches, so-called “microglial nodules,” endowed with a pervasive pro-inflammatory effect that extends throughout the brain.

To decipher the underpinnings of neurological symptoms, Song et al. (33) performed single-cell transcriptomics on immune cells from the CSF and blood of neuro-COVID-19 patients and healthy donors. They found and increased activation of CSF T cells, that strongly interacted with overactive NK and dendritic cells, displaying upregulation of IL-1 and IL-12 pathways and suggesting a coordinated and compartmentalized T-cell based response to CNS antigens. Also, B cells were enriched in COVID-19 CSF compared with controls, and their antibody profile differs from peripheral blood. A subset of intrathecal antibodies—especially anti-Spike—also targets neural antigens. A similar approach reported autoantibodies targeting known antigens (Yo, NMDA receptors) and undetermined epitopes of myelin, endothelium, astrocytes, and neurons, some of which help explain clinical features (e.g., seizures) (34).

SARS-CoV-2 is associated to a growing number of para-infectious and postinfectious neurological conditions that have an immunological substrate (35–38): in particular, Acute Disseminated Encephalomyelitis (ADEM) results extremely frequent (39). Para-infectious ADEM occurrence often correlates with COVID-19 severity, suggesting off-target effects of the hyperinflammatory response involving brain tissue and vasculature (40). Indeed, beyond deep white matter demyelination, these cases often display intraventricular and intraparenchymal hemorrhages (e.g., microbleeds), with features of fulminant necrotizing encephalitis (41–44). Conversely, post-COVID-19 ADEMs appear unrelated to respiratory involvement and do not differ from those triggered by other infections, also in terms of prognosis (45, 46). Interestingly, COVID-19 was shown to trigger an exacerbation of a recent, but remitted, Coxsackie virus–induced ADEM (47). It was also suggested that SARS-CoV-2-associated ADEMs have an atypically frequent involvement of spine—especially spinal gray matter (48).

Literature reviews identified 43 cases of Acute Transverse Myelitis (49) reported until January 2021 in association with COVID-19. As the first reports of neurological complications of SARS-CoV-2 infections did not involve myelitis, such a number of cases (with an estimated incidence of 0.5 per million) resulted somehow unexpected. Of interest, no association has been made between ATM and the other highly virulent beta-coronaviruses, suggesting a special property of SARS-CoV-2 in triggering spine demyelination. Patients’ age ranged from 21 to 73 years, male sex slightly prevailed, and COVID-19 course was prevalently mild. A third of cases developed neurological impairment within 5 days from the onset of COVID-19 symptoms, compatible with para-infectious physiopathology; however, the greatest number of cases represent postinfectious dysimmune complications, with a maximum latency of 6 weeks. Twenty percent of patients had myelitis in the context of an ADEM, which instead involved mostly women. Longitudinally extensive lesions (as in Longitudinally Extended Transverse Myelitis, LETM) involving >3 spinal cord segment appeared typical, occurring in 70% of COVID-19-associated myelitis cases; in a 28-year old woman, demyelination involved the entire spine, from the medulla oblungata to the conus medullaris (50).

More recently, additional and, to some extent, peculiar CNS demyelinating events linked to COVID-19 have been reported. Zoghi et al. (51) described a case of a 21-year-old male that developed encephalomyelitis 2 weeks after mild COVID-19. At admission, he manifested a confusional state with recurrent vomit, paraparesis, and a T8 sensory level. MRI revealed scarcely enhancing FLAIR hyperintensities extending from internal capsules to the cerebral peduncles and pons, marbled-pattern hyperintensities in corpus callosum, and a longitudinally extensive transverse myelitis in the cervical and thoracic spine. CSF analysis showed pleocytosis, hypoglycorrhachia, and hyperprotidorrhachia, without oligoclonal bands (OCBs). Image findings and clinical onset (alike area-postrema syndrome) pointed toward a possible NMOSD, but Aquaporin-4 receptor (AQP4) and myelin oligodendrocyte glycoprotein (MOG) antibodies in the serum and CSF were absent. Treatment with plasma exchange only provided partial recovery. The case was framed as an atypical ADEM attributable to a postinfectious dysimmune state. A more stereotypical acute anti-MOG CNS syndrome following SARS-CoV-2 infection had been previously described by Zhou et al. (52) characterized by bilateral, sequential, severe optic neuritis with contemporary involvement of the cervical and thoracic spine. The finding of anti-AQP4 antibodies in CSF and serum in a case of post-COVID-19 encephalomyeloradiculitis also suggests a nexus between SARS-CoV-2 and neuromyelitis optica spectrum disorder (NMOSDs) pathogenesis (53, 54)

Rodriguez de Antonio et al. (55) reported the case of a 40-year-old woman with asymptomatic SARS-CoV-2 infection that developed, in the late phase of a totally asymptomatic SARS-CoV-2 infection, hypoesthesia and bladder dysfunction. MRI revealed a T5-6 lesion; serological essays found the presence of IgM anti-gangliosides (GD2 and GD3), more commonly seen in peripheral nervous system inflammatory disorder (such as Guillain Barrè Syndrome, GBS). Indeed, IgM anti-GD1b were detected in a post-Covid Acute Motor Axonal Neuropathy (AMAN) variant of GBS (56). However, whether SARS-CoV-2-induced demyelination is endowed with distinct features and biomarkers should be clarified.

## COVID-19 *vs* MS: Immunopathogenetic Crossroads

The above-mentioned immunological pathways dysregulated in COVID-19 largely overlap with MS pathogenetic mechanisms and suggest that SARS-CoV-2 infection could act as an environmental risk factor for disease manifestation in susceptible individuals. This hypothesis is reinforced by clinical and experimental studies that unravel host-pathogen interactions underlying COVID-19 at both virological and genetic levels, intersecting those dealing with the major components of MS etiopathogenesis.

Based on the principle that genetic data inform on multifactorial disease pathogenesis, a system biology study found significant interactions of SARS-CoV-2 with genes associated with autoimmune diseases and comorbidities that predispose to a severe COVID-19 course. The strong association with autoimmunity was peculiar of SARS-CoV-2 with respect to other Coronaviruses and respiratory viruses, in line with the exuberant and autoaggressive immune response that causes COVID-19. Interestingly, MS-associated genes were the most enriched in SARS-CoV-2 host’s interactors, suggesting pathophysiological overlaps that are worth investigating (57). In particular, there are three pivotal crossroads of MS and COVID-19 immunological substrates: the type-1 IFN (IFN-I) response, the TH-17 axis, and the inflammasome pathway.

In MS patients, an impairment in IFN-I response has been widely described at the genetic and transcriptional levels and is confirmed by three decades of successful treatment with IFN-1B formulations (58, 59). Studies on severe COVID-19 patients highlighted a defective IFN-I production, with a consequent delayed and unrestrained T cell response, enhancing both viral spread and cytokine release that predispose to multiorgan failure (60, 61). Notably, the use of beta-interferons decreases the risk of COVID-19 in people with MS (pwMS) (62). Coronaviruses can interfere with IFN-I production and actions through multiple non-structural proteins. It has been demonstrated that SARS-CoV-2 antagonizes IFN-I secretion and signaling more efficiently than SARS-CoV-1 and MERS-CoV (63). These intrinsic viral properties are detrimentally boosted if the host itself displays a blunted IFN system: in severely ill COVID-19 patients, Zhang et al. found an enrichment in genetic variants accounting for an impairment of IFN-I immunity (64), while Bastard et al. reported augmented levels of IFN-I neutralizing antibodies (65). These data may suggest that people at risk for developing MS (just as pwMS not receiving DMTs) may be more susceptible to the virus-induced dysimmune effects; likewise, the repurposing of IFN-beta from MS armamentarium for the fight against COVID-19 has a biological rationale if administered early after the infection, as an exogenous “supplement” to aid viral suppression (35, 66).

Th1 cells producing IFN-gamma and Th17 cells secreting IL-17 are the principal T lymphocyte subsets involved in MS pathogenesis, to the point that most immunomodulating therapies aim to restore immunological balance interfering with their induction and skewing towards the Th2 axis (2). Interest in Th17 cells in MS is growing, since their discovery in 2005. They are induced by IL-6 produced by pathogenic B cells, contribute pleiotropically to CNS inflammation, and are the principal player of the brain autoimmune attack induced by intestinal microbiota alteration in pwMS (36–38). Severe COVID-19 cases exhibit high levels of IFN-γ and IL6 and a skewing towards Th17 cells (67, 68); moreover, IL-17 levels correlate with clinical severity, suggesting the opportunity of therapeutically inhibiting the Th17 axis (69). A sophisticated virus-host interaction analysis discovered that IL-17 receptor A (IL17RA) physically interacts with SARS-CoV-2 Orf8 protein, and highlighted a genetic locus associated with COVID-19 severity whose biological effects relate to IL-17 actions (70). Also, SARS-CoV-2 is capable of infecting enteric cells (71), and microbiota alterations correlate with clinical outcomes (72), so that the contribution of a gut-lung axis including Th17 cells in COVID-19 has been postulated (73). These elements make likely the possibility that Th17 axis could be involved in SARS-CoV-2 effects in pwMS.

Inflammasomes are cytosolic multimeric complexes driving the inflammatory response of the innate immune system. They activate Il-1Beta and IL-18 upon sensing of endogenous (tissue damage) or exogenous (e.g., infections) stressors by pattern-recognition receptors (PRRs), such as NLRP3. In MS, NLRP3-inflammasome significantly contributes to the chronic inflammation led by microglial cells that propels neurodegeneration. *IL1B* gene is upregulated in blood and brain lesions of primary progressive MS patients (74) due to an NLRP3 overactivity, on which IFN-I may interfere (75). Moreover, gain-of-function variants in NLRP3 and IL1B genes correlate with severity and progression of MS (76). Since the SARS-CoV-1 E, ORF3a, and ORF8b proteins directly activate NLRP3-inflammasome, similar effects are hypothesized in SARS-CoV-2. An increased potency of ORF3a-NLP3 interaction may underly the augmented virulence of the novel coronavirus (77–79). Being the activation of “danger sensors” among the early activators of an immunopathogenetic process, it is plausible that the immune response following SARS-CoV-2 infection could contribute to neuroinflammation in genetically susceptible people, precipitating MS onset or even impacting on progression (80).

## SARS-CoV-2 and MS-Associated Viruses: Partners in Crime?

Indirect mechanisms through which SARS-CoV-2 infection may interfere with MS course may be sustained by the interactions with long-studied MS-associated pathogens: the *Herpesviridae* members Epstein Barr Virus and Human Herpes Virus 6 (HHV-6), as well as the Human Endogenous Retroviruses (HERVs).

A longstanding, quasi-causal evidence supports the Epstein-Barr Virus (EBV) as a necessary environmental interactor (81) involved across the whole spectrum of MS (82). High levels of antibodies against EBV antigens (EBNA1 and VCA) at least triplicate the risk of developing MS; a history of infectious mononucleosis (IM), the clinical syndrome caused by a post-childhood EBV infection, exerts a similar effect (83, 84). The prevalent hypothesis to explain these sero-epidemiological evidences is an augmented immune reactivity reflecting insufficient control of viral infection, due to EBV-specific T lymphocyte exhaustion or scarcity: the quasi-persistent active state in periphery and CNS promotes neuroinflammation and disease progression (85–90).

Lymphopenia and T cell exhaustion, which characterize COVID-19, may impair the control of EBV latency in B cells [also within meningeal follicles (86)], favoring viral reactivation, rising up anti-EBV titers and exacerbating the hyperinflammatory state. Indeed, severe COVID-19 cases display higher prevalence and levels of EBV viremia compared with non-COVID critically ill subjects; the virus’s DNA titers and anti-EBNA1 IgM levels correlate with increased inflammatory markers (C-reactive Protein, IL-6), disease severity, and prognosis (91–93). Paolucci and colleagues screened for opportunistic viral infections 104 moderate to severe COVID-19 cases: EBV was the only detected (95.2% of the ICU patients and in 83.6% Sub-ICU patients). Reactivation correlated with reduction of CD8+ T and NK lymphocyte count, supporting the hypothesis of a defective cell-mediated control (94).

A SARS-CoV-2 -EBV interaction may extend beyond the acute phase and play a role in the pathogenesis of “long-COVID,” or post-COVID-19 syndrome. It manifests with lingering symptoms and/or delayed or long-term complications beyond 4 weeks from the acute phase. Most patients suffer of fatigue or muscle weakness, sleep difficulties, and anxiety or depression, but organ-specific sequelae also play a part (94, 95). Neuropsychiatric involvement appears to be typical and frequent enough that a more specific qualifier of “Post-COVID-19 Neurologic Syndrome” (PCNS) has been coined (96, 97). Gold and colleagues found that two-thirds of COVID-19 long-haulers had EBV reactivation and speculated this might contribute to clinical manifestations.

An integrated, molecular portrait of post-COVID-19 syndrome pathogenesis is still lacking; however, in patients’ and clinicians’ descriptions of this heterogeneous condition, similitudes with Myalgic Encephalomyelitis/Chronic Fatigue syndrome (CFS) stand out (98). This syndrome comprehends extreme physical and cognitive impairment that are typically preceded by an acute infectious disease: EBV and HHV-6 A/B have been historically implicated (99–102). Mechanisms contributing to its pathogenesis include immune exhaustion and dysregulation subsequent to an exuberant acute response to the pathogen (increased cytokines, presence of autoantibodies), a multisystemic hypometabolic state with redox imbalance, intestinal dysbiosis, and diffuse neurological dysfunction (pain hypersensitivity, subtle CNS neuroinflammation with glial cell dysmetabolism, impaired brain connectivity) (103–106). Fatigue concerns more than 75% pwMS during disease course—including the prodromic phase (107)—and is frequently considered as the worse symptom (108, 109). Nonetheless, it is often overlooked by clinicians, also because no drug has proven to be effective in tackling it. A recent randomized clinical trial demonstrated no difference between placebo and three commonly prescribed symptomatic medications (amantadine, modafinil, methylphenidate) in easing MS-related fatigue (110). Beyond clinical similarities—also mirrored by neuroimaging functional studies (111)—there are significant molecular overlaps between CFS and MS, as objectively assessed by immunological studies (112–114). A notable example is the high frequency of circulating CD8+ mucosal-associated invariant T cells (MAIT) in both CFS and MS patients (115), where they bridge intestinal dysbiosis to CNS inflammation (116–118). Given the novelty and the expected high prevalence of post-COVID-19 syndrome, research on CFS and MS-associated fatigue pathomechanisms will hopefully gain momentum, paving the way toward targeted therapies for these conditions.

EBV reactivation could be as a simple byproduct of COVID-19 unbalanced immunity; however, data are emerging that show probable virus-virus synergies at the expense of the host. An *in-silico* analysis by Vavougios (119) dissected overlapping host gene signatures between SARS-CoV-2 and other co-pathogens. EBV-induced expression pattern was the most significantly enriched, suggesting a mechanistic interplay between the two viruses operating at the host’s transcription level. The interaction nodes shared by the two viruses include Heat-Shock Proteins (HSPA8, HSPA5, HSPD1, etc.), ribonucleoproteins, and post-translational regulators (SYNCRIP, SERBP1, SSBP1).

EBV may also enhance SARS-CoV-2 infectivity. The expression of ACE2, the principal SARS-CoV-2 surface receptor, is regulated by EBV’s ZTA transcription factor and peaks when EBV enters the lytic replicative cycle in epithelial cells. Its reactivation proved to facilitate ACE2-dependent coronavirus infection on pseudovirus assays (120). Neuropilin-1 (NRP1) has been recognized as a coreceptor of SARS-CoV-2, binding the Spike protein after its cleavage by proteases (121, 122). Interestingly, NRP1 also facilitates EBV cell entry, and its interaction with EBV-gB protein activates intracellular kinase pathways resultant in a positive feedback on viral infectivity (123). This transmembrane protein is expressed in various immune cells. Most of studies focused on Tregs, whose action ends in the modulation of Th1 and Th17 subsets (124, 125). Deletion of NRP1 exacerbates Experimental Autoimmune Encephalitis, the most used murine model of MS (124). Moreover, its expression is augmented in endothelial cells of active MS lesions (125) in response to the blood-brain barrier (BBB) disruption.

Alike to EBV, HHV6 is a neurotropic and lymphotropic pathogen that persists lifelong in the host as an episome or through chromosomal integration in glial cells. High titers of anti-HHV6 antibodies are epidemiologically associated to MS development risk, both alone and by interacting with anti-EBNA1 titers (126). Several HHV-6 pathogenetic mechanisms have been pinpointed in MS: enhancing on-site inflammation in MS lesion, mimicry toward myelin antigens, direct provocation of apoptosis of glial cells and consequent epitope spreading, and induction of neuronal death due to glutamate excitotoxicity (10).

Evidence on HHV-6 reactivation during SARS-CoV-2 infection is less solid, and the Herpesvirus is mainly associated to COVID-19 dermatological manifestations (127). Nonetheless, a neurological event associated to SARS-CoV-2/HHV6 interaction was described by Jumah et al. (128): a 61-year-old man experiencing lymphopenia and hypogammaglobulinemia during COVID-19 developed a para-infectious acute myelitis. CSF analysis showed a high HHV-6 viremia, and anti-MOG antibodies were detected in serum. In this case, the two viruses—individually linked to MOG-associated diseases (52, 129)—might have synergized in triggering immune-mediated demyelination. A direct interaction alike to that seen for HHV-6A with EBV in MS is therefore conceivable (130).

HERVs result from the integration of ancient exogenous retroviruses into human genome. Their transcription is finely regulated through epigenetic mechanisms and particularly affected by other viruses’ infection, including EBV and HHV-6. Among HERVs, HERV-W has been linked to MS pathogenesis thus being also named MS-Related Virus (MSRV). Notably, HERVs are also associated to CFS (131). When expressed, HERV-W Env protein acts as a superantigen, activates CNS-oriented T lymphocytes and microglia, sustaining a proinflammatory state and impairing myelin homeostasis (10). These mechanisms translate to MS relapses and progression, to the point that MSRV proteins have been candidate as biomarkers and therapeutic targets in MS (132, 133).

Charvet and colleagues demonstrated that SARS-CoV-2 activates HERV W and HERV-K *Env* transcription *in vitro* through the interaction of Spike with ACE2 on T lymphocytes (134). Accordingly, *ENV* is strongly expressed in blood cells of COVID-19 patients but not in healthy donors, and its level correlates with inflammatory mediators (IL-6, IL-17, and CXCR1), and with markers of T-cell exhaustion (e.g., PD1). Being predictive of a severe prognosis, *ENV* expression could play a role in immune overactivation and contribute to acute and chronic COVID-19 complications (135). In this context, HERVs could act as endogenous modulators of SARS-CoV-2/host interaction, helping to explain the extreme inter-individual variability in COVID-19 manifestations (136). Along this line, potential interplays of Herpesviruses and HERVs with SARS-CoV-2 might also be conceivably supposed to impact on MS pathogenesis and affect disease course.

## COVID-19 and SARS-CoV-2 Vaccines in People With Multiple Sclerosis: What We Know

Relevant to our discussion is the possible role of SARS-CoV-2 infection in unveiling definite MS. To date, four cases have been described in literature, in which, during or post COVID-19, neurological syndromes in young people (three women, one man) appeared as typical onset of MS fulfilling McDonald’s 2017 criteria (**Table 1**). Such event cascade may be (a) purely casual, *i.e*., MS onset occurred by chance after SARS-CoV-2 infection; (b) non-specific, *i.e.*, COVID-19 denotes the increase morbidity observed in people that will in a few years develop MS—so-called “MS prodrome” (141); (c) specific, *i.e.*, SARS-CoV-2 infection acts as an environmental risk factor for MS. Of course, given the youth of the novel Coronavirus, evidence is insufficient to take conclusions, and large-scale, case-control studies are awaited to answer.

**Table 1:** Reported cases of MS onset in a temporal relationship with COVID-19 .

Reference	Patient characteristics	Timing	COVID-19	Long-COVID-19	MS onset	MRI lesions	CSF
Palao et al. (137)	29-year-old woman	After 2–3 weeks	Mild (Anosmia, dysgeusia, myalgias)	–	Optic neuritis, pyramidal signs	1 CE optic nerve 1 CE right occipital lobe 1 non-enhancing left temporal lobe	OCBs+
Fragoso et al. (138)	27-year-old woman	After 6 months	Mild (fever, cough, anosmia, hypogeusia)	Persistent anosmia	Sensory loss, pyramidal signs	1 CE frontal 1 CE cervical lesion 3 non-enhancing periventricular	OCBs +
Moore et al. (139)	28-year-old man	After 10 days from symptoms onset	Mild (Anosmia, sore throat, cough, myalgias, headache)	–	Internuclear ophthalmoplegia	1 CE right parietal lobe 1 CE right superior cerebellar peduncle 1 CE right middle cerebellar peduncle >3 non-enhancing periventricular, corpus callosum	OCBs (n=5)
Moghadasi A.N (140)	31-year-old woman	Concomitant	Mild (anosmia, ageusia)	–	Optic neuritis	>3 non-enhancing periventricular and pericallosal	OCBs+

CE, contrast-enhancing; OCBs, oligoclonal bands.

The immune activation consequent to a microbial infection often embraces some grade of non-specific systemic inflammation that is known to enhance autoimmunity in people with MS (pwMS) and interfere with the disease course (142–144). Upper respiratory tract infections (URTI), typically caused by adenoviruses, rhinoviruses, flu viruses, and coronaviruses, have been extensively proven to trigger both clinical and radiological MS relapses (145–147). Moreover, Edwards et al. demonstrated that a serological confirmation of URTI corresponded to a threefold augment in MS exacerbation risk (145). Precisely, McDonald’s criteria define *relapse* as “a monophasic clinical episode with patient-reported symptoms and objective findings typical of multiple sclerosis, reflecting a focal or multifocal inflammatory demyelinating event in the CNS, developing acutely or subacutely, with a duration of at least 24 hours, with or without recovery, and in the absence of fever or infection” (148). Conversely, the term *pseudo-*relapse refers to recrudescence of previously experienced MS symptoms concomitant to an elevation of body temperature, as during physical activity or infection-related fever. A *pseudo*-relapse lasts more than the brief, self-limiting exacerbation of a focal deficit typical of the Uhthoff’s phenomenon induced by heat: thus, it is easily distinguished from common day-to-day fluctuations of chronic symptoms, but the discrimination from relapses is more challenging (149).

Emerging data show that SARS-CoV-2 infection can trigger both *pseudo*-relapses and true relapses. A large community-based study by Garjani et al. (150) showed that the majority of pwMS developing COVID-19 reported clinical worsening of pre-existing neurological symptoms, and 20–35% developed new MS symptoms, mostly motor or sensory in nature, that could last for months following the acute infectious phase (thus being considerable as *true* relapses). These attacks were referred as worse than those experienced with non-COVID-19 infections. Accordingly, a smaller study from New York University MS Centre reported a 21.1% recrudescence rate (151). DMTs appear to be protective towards SARS-CoV-2-induced relapses. Indeed, pwMS receiving DMTs are less likely to develop new MS symptom during COVID-19 compared to untreated patients (152). On the other hand, a possible “protective” effect of COVID-19 on relapse rate was suggested by Etemadifar and colleagues (153), who conducted a retrospective study on 125 relapsing-remitting pwMS, 56 of whom had laboratory confirmed SARS-CoV-2 infection. Only 7.14% of pwMS in the COVID-19 group experienced a clinical relapse compared with 26.09% of the non-COVID-19 group. The authors hypothesized that COVID-19-associated lymphopenia could have a role in containing the proliferative rate of CNS-targeting T clones. However, no significant difference emerged in a self-comparison assessment of each group: relapse rate of each patient was similar for comparable windows taken before and during the pandemic (i.e., after SARS-CoV-2 infection, for the COVID-19 group). This suggests a possible selection bias: control group included *(i)* pwMS with an intrinsically higher relapse rate and/or (*ii*) pwMS receiving mostly first-line, less effective DMTs—which, on the other side, may have reduced their infection risk.

Indeed, since the beginning of the pandemic, research efforts have focused on answering urgent questions: (i) Are pwMS more susceptible to SARS-CoV-2 infection? (ii) What is the clinical course of COVID-19 in pwMS? (iii) What is the impact of DMTs on COVID-19 susceptibility and prognosis? After the first pioneering Italian observational study (62, 154), many others provided converging results that supported clinical practice and reassured both neurologists and patients (155). Besides some discordances among results (mostly imputable to heterogeneity in cohorts’ characteristics and data collection), preliminary findings have been corroborated, thanks to larger samples’ numerosity and pooled analyses (62), allowing to reasonably draw some conclusions. First, pwMS do not appear to have an increased risk of contracting COVID-19. Older age, male sex, and the presence of comorbidities negatively affect the infection’s clinical course in pwMS as in non-MS people. Except for fatigue, which is expectedly more frequent in pwMS, respiratory and systemic symptoms did not differ between pwMS and the general population. Neurological disability and progressive disease course emerged as linked to COVID-19 severity, consistent with a frail status of this subgroup of patients.

In general, DMTs showed an acceptable level of safety with respects to SARS-CoV-2 infection outcomes. Type I IFN formulations showed a protective effect (in line with the role of IFNs in restraining SARS-CoV-2 in the early infectious phase). Instead, a complicated course of COVID-19 occurred more frequently in the following circumstances: pwMS not receiving any DMT, cases under therapy with anti-CD20 agents (ocrelizumab, rituximab), or people who get infected soon after receiving high-dose corticosteroids (62, 155).

Present knowledge reflects the pandemic scenario during the first and second waves of SARS-CoV-2 spread. The emergence of variant of concern, potentially endowed with augmented infectivity and virulence (156), emphasizes the need of continuing clinical and epidemiological monitoring of pwMS. Variant selection and the overall pandemic trend largely depend on ongoing vaccination campaigns, which have started in late 2020, after the speed approval of mRNA-based (Pfizer and Moderna) and adenovirus-vectored formulations (AstraZeneca and Johnson & Johnson’s). Since then, focus has shifted to ascertain safety and efficacy of the anti-COVID-19 vaccines in pwMS.

Trials and real-life experiences with diverse “traditional” vaccine formulations (influenza, tetanus, meningococcus, etc.) showed that active immunization is safe in pwMS. While no specific issues exist for non-live formulations, administration of live-attenuated vaccines (such as VZV) should be programmed before starting DMTs and avoided during immunosuppression owing to the risk of a productive infection (152, 157). A satisfactory level of safety has been promptly confirmed for anti-COVID-19 mRNA-based vaccines: in a study by Achiron et al. (158) on 555 pwMS receiving Pfizer-BioNTech vaccine, frequency and characteristics of vaccine-induced adverse events were almost comparable with non-MS population and more numerous in young people; in a cohort of 239 pwMS surveyed by Lotan and colleagues (159), reactions were even less common. Detailed studies on mRNA-1273 and adenovirus-based vaccines’ safety in pwMS are lacking and awaited to consolidate clinical indications.

In general, most studies have ruled out an increased long-term risk of developing MS (160) as well as a short-term risk of neurological relapse in people with clinically defined MS (161) following vaccinations. However, some caution should be taken when vaccinating pwMS with sustained disease activity and/or a recent clinical or radiological relapse, in which the threshold for inflammatory exacerbation concomitant to the immunization stimulus could be lower (162–165). The same could be hypothesized for people with subclinical CNS inflammation (such as in radiological isolated syndromes, RIS) or genetically at-risk individuals during disease prodrome. Havla et al. (166) described the case of a 28-year-old woman with a familiar history of MS who developed a sensorimotor syndrome 6 days after the first administration of BNT162b2 SARS-CoV-2 vaccine. MRI revealed an active myelitis and non-enhancing, disseminated white matter lesions in the brain; OCBs were present in the CSF, and a diagnosis of MS was made. Early data on cohort BNT162b2 vaccination have not highlighted an increased risk of relapse in the very-short term (158); in another study on the same vaccine, 15.1% of pwMS reported worsening or new neurological symptoms that lasted more than 3 days in 55.6% of cases (159). However, further investigation on the long term is warranted to establish whether anti-COVID-19 vaccines’ safety profile is confirmed in MS.

Concerning vaccine efficacy, studies are ongoing that evaluate serological response as a surrogate of protection against COVID-19 in pwMS. It is known that some DMTs can negatively affect active immunization. Evidence recently reviewed in (167) indicates that fingolimod, natalizumab, alemtuzumab, and the anti-CD20 monoclonal antibodies (ocrelizumab, rituximab) may reduce serological response to “traditional” vaccines (inactivated, conjugate, toxoid). As presumable and in line with studies on rheumatic diseases, anti-CD20 appears to have the greatest impact on antibody production, which is partly related to the levels of circulating B cells at the time of administration (168). Before the pandemic, no studies had been conducted on vaccination efficacy in pwMS receiving the oral B-depleting agent cladribine. Converging results are coming from studies assessing humoral response following natural SARS-CoV-2 infection and vaccinations. Indeed, following COVID-19, lower anti-SARS-CoV-2 IgG titers were found in pwMS receiving fingolimod or anti-CD20, the being latter associated to a completely absent response (169–172). Similar results are coming from observational studies evaluating post-vaccination COVID-19 serology **(**displayed in **Table 2** **).** Fingolimod may attenuate (if not abate) humoral response, especially in pwMS with reduced lymphocyte count at the time of administration; such response is frequently absent in pwMS receiving ocrelizumab or rituximab, and when present, it depends on the time interval between the last drug infusion and vaccination. Other DMTs, including cladribine, did not appear to interfere with post-COVID-19 or post-vaccination serological response. Interestingly, the largest study to date evidenced a significantly higher humoral response to mRNA-1273 vaccine compared to BNT162b2, suggesting its preferential use in pwMS at risk of unsuccessful immunization (179).

**Table 2:** Studies on COVID-19 vaccination in people with MS (until July 31^st^, 2021).

Reference	Number of pwMS (female)	Vaccine	Serological assay	Meaning
Bigaut et al. (173)	28 (23)	Pfizer-BioNTech (BNT162b2) Moderna (mRNA-1273)	Abbott or Roche SARS-CoV-2 IgG assay (spike protein), U/ml	Lower IgG titers in patients treated with anti-CD20 and in patients treated with fingolimod compared to patients receiving other DMT or untreated pwMS
Gallo et al. (174)	4 (1)	Pfizer-BioNTech (BNT162b2)	LIAISON^®^SARS-CoV-2 TrimericS-IgG assay (DiaSorin-S.p.A.), BAU/ml	Quasi-negative serology in four pwMS treated with ocrelizumab as compared to 55 healthy subjects.
Achiron et al. (175)	125 (72)	Pfizer- BioNTech (BNT162b2)	EUROIMMUN (EI, Lubeck, Germany) anti-SARS-CoV-2 IgG quantitative ELISA	Negative serology in pwMS treated with fingolimod, irrespective to absolute lymphocyte count Positive serology in only 22.7% pwMS treated with anti-CD20 Untreated pwMS and pwMS receiving Cladribine had igG titers similar to healthy subjects
Buttari et al. (176)	4 (4)	AstraZeneca (AZD1222) Pfizer-BioNTech (BNT162b2)	unknown	Positive serology in 2/2 pwMS receiving Cladribine Negative serology in 1/2 pwMS receiving Ocrelizumab (vaccinated 2 months after the last infusion)
Guerreri et al. (177)	32 (22)	Pfizer-BioNTech (BNT162b2) Moderna (mRNA-1273)	*ECLIA* electrochemiluminescence immunoassay, *CLIA* chemiluminescence immunoassay, *CMIA* chemiluminescence microparticle immunoassay	Positive serology in 62.5% pwMS receiving fingolimod and 37.5% pwMS receiving Ocrelizumab
Drulovic et al. (178)	22 (17)	Pfizer-BioNTech (BNT162b2) Beijing/Sinopharm (BBIBP-CorV)	*ELISA SARS-CoV-2 IgG (INEP, Belgrade, Serbia)*	Positive serology in 100% pwMS treated with cladribine and vaccinated with BNT162b2 Positive serology in 42.9% pwMS treated with cladribine and vaccinated with BBIBP-CorV Positive serology in 100% pwMS receiving alemtuzumab and vaccinated with BNT162b2 or BBIBP-CorV Time since last administration and absolute lymphocyte count did not affect serological response
Sormani et al. (179)	780 (517)	Pfizer-BioNTech (BNT162b2) Moderna (mRNA-1273)	*Electrochemiluminescence immunoassay (ECLIA) (Elecsys^®^, Roche Diagnostics Ltd, Switzerland).*	Positive serology in 92.9% pwMS on fingolimod, 43.5% on ocrelizumab, 64% on rituximab Lower IgG titers in patients receiving ocrelizumab fingolimod and rituximab In pwMS receiving anti-CD20, antibody levels correlate to the interval from the last infusion to vaccine administration In pwMS receiving fingolimod, lymphopenia is associated to lower antibody levels Vaccination with mRNA-1273 elicited 3.5-fold higher serological response than with the BNT162b2 vaccine.`

Beyond humoral response, a critical role in adaptive immunity to SARS-CoV-2 is exerted by cytotoxic and helper T cells. As quantitative correlates of serological protection are still unclear, assessing SARS-CoV-2-specific T-cell response in naturally infected or vaccinated subjects is of increasing importance, given that emerging viral variants evading humoral neutralization do not escape cellular responses (180, 181). They appear even more relevant when antibody production is substantially dampened, as in the case of B-cell-depleted pwMS. To this regard, preliminary evidence showed that the majority of pwMS receiving Ocrelizumab who experienced COVID-19 were able to mount an efficient and T-cell response toward SARS-CoV-2 (182). Apostolidis and colleagues (183) performed an extensive immunological study on 20 B-cell depleted pwMS (19 on Ocrelizumab and 1 on Rituximab) following mRNA SARS-CoV-2 vaccination. Besides confirming a significant impairment in antibody and memory B cell responses, they found a robust T cell response, with SARS-CoV-2 specific CD8+ expansion exceeding that of healthy controls, especially in pwMS completely lacking igG production. These findings are in line with some reported cases of favorable course of COVID-19 in pwMS receiving anti-CD20 (184, 185) and reassure about the efficiency of vaccination in immunocompromised patients. Similar immunological investigations are awaited in pwMS receiving other DMTs, in order to get an integrative view on vaccine-induced protection against COVID-19 to optimize immunization strategies.

To sum up, current evidence favors recommending administration of SARS-CoV-2 vaccines to pwMS, as the benefit of protection from COVID-19 largely outweighs the potential risks; timing with respect to DMTs and type of vaccine should be tailored case by case by neurologists, taking into account the clinical history and additional risk factors (186).

## Conclusions

Almost 2 years of SARS-CoV-2 pandemic have profoundly impacted on medical world and scientific research, including the field of MS. We believe that the “research rush” triggered by the novel Coronavirus may propel studies on MS; *vice versa*, deepening our knowledge on MS pathogenesis and its relationship with viral infections could guide the investigations on COVID-19 immunopathogenesis.

Establishing such a virtuous cycle may be relevant to multiple aims. Technological advances rapidly mastered to deal with SARS-CoV-2 could be translated soon to address MS issues: a prominent example comes from the potential application of mRNA-based technology to design tolerizing vaccines that dampen CNS autoimmunity (187). Conversely, some MS DMTs could be repurposed to manage COVID-19-associated immune dysregulation. Clinical trials are already ongoing to evaluate IFN-B, fingolimod, dimethylfumarate; additionally, masitinib (a tyrosine-kinase inhibitor that showed efficacy in phase 2B/3 trials for progressive MS) has been recently highlighted as a potent Coronavirus inhibitor (188).

Furthermore, pinpointing the biological substrates of neuroinflammatory events associated with COVID-19 could help extend knowledge on MS-associated viruses in the etiopathogenesis of both conditions. The possibility that SARS-CoV-2-induced CNS autoimmune syndromes represent a distinct nosological entity should be evaluated through long-term studies (189); likewise, large case-control studies are needed to test a possible association between SARS-CoV-2 infection and MS development. A long follow-up of pwMS that suffered of COVID-19 will evaluate a potential inference in disease natural history, as well as the frequency and manifestation of long-COVID-19 in these patients. To this aim, it will be crucial to perform thorough immunological investigations on biological samples along with epidemiological studies evaluating past SARS-CoV-2 infection or vaccination as clinical variables.

In its transition to endemicity, COVID-19 is expected to become a mild disease of the childhood (190). Will a post-adolescence infection, repeated infection, or vaccine-induced immunity reshape the future epidemiology of MS? While dealing with the unprecedented scenario of a newborn virus pandemic, we might find hints to improve our understanding on this complex disorder.

## Author Contributions

GB conceived the idea and wrote the manuscript. GB, VR, MB, RR, RB, GP, EM, CR, AM, and RM performed bibliographic search and contributed to data interpretation. GR and MS reviewed the manuscript. All authors contributed to the article and approved the submitted version.

## Funding

MS and GR are supported by CENTERS, a Special Project of, and financed by, FISM—Fondazione Italiana Sclerosi Multipla.

## Conflict of Interest

MS received research support and consulting fees from Biogen, Merck, Novartis, Roche, Sanofi, Teva.

The remaining authors declare that the research was conducted in the absence of any commercial or financial relationships that could be construed as a potential conflict of interest.

## Publisher’s Note

All claims expressed in this article are solely those of the authors and do not necessarily represent those of their affiliated organizations, or those of the publisher, the editors and the reviewers. Any product that may be evaluated in this article, or claim that may be made by its manufacturer, is not guaranteed or endorsed by the publisher.
